# Estimation of Relative Hand-Finger Orientation Using a Small IMU Configuration

**DOI:** 10.3390/s20144008

**Published:** 2020-07-19

**Authors:** Zhicheng Yang, Bert-Jan F. van Beijnum, Bin Li, Shenggang Yan, Peter H. Veltink

**Affiliations:** 1 Department of Biomedical Signals Systems, Technical Medical Centre, University of Twente, 7500 AE Enschede, The Netherlands; b.j.f.vanbeijnum@utwente.nl (B.-J.F.v.B.); P.H.Veltink@utwente.nl (P.H.V.); 2 School of Marine Science and Technology, Northwestern Polytechnical University, Xi’an 710072, China; libin_cme@nwpu.edu.cn (B.L.); yshgang@nwpu.edu.cn (S.Y.)

**Keywords:** relative orientation estimation, IMU, magnetometer-free

## Abstract

Relative orientation estimation between the hand and its fingers is important in many applications, such as virtual reality (VR), augmented reality (AR) and rehabilitation. It is still quite a big challenge to do the estimation by only exploiting inertial measurement units (IMUs) because of the integration drift that occurs in most approaches. When the hand is functionally used, there are many instances in which hand and finger tips move together, experiencing almost the same angular velocities, and in some of these cases, almost the same accelerations are measured in different 3D coordinate systems. Therefore, we hypothesize that relative orientations between the hand and the finger tips can be adequately estimated using 3D IMUs during such designated events (DEs) and in between these events. We fused this extra information from the DEs and IMU data with an extended Kalman filter (EKF). Our results show that errors in relative orientation can be smaller than five degrees if DEs are constantly present and the linear and angular movements of the whole hand are adequately rich. When the DEs are partially available in a functional water-drinking task, the orientation error is smaller than 10 degrees.

## 1. Introduction

Hand-finger movement tracing is useful in many areas, such as virtual reality (VR), augmented reality (AR), ergonomic assessment and especially medical applications [1,2,3,4,5]. People who suffered from stroke or injury of the spinal cord need an effective rehabilitation therapy for recovery of body functions, including hand function. In a hospital, therapists evaluate the hand function through some traditional assessments such as the Fugl–Meyer or Jebsen–Taylor hand function assessment [6,7]. Currently, the results may be subjective and dependent on the therapist. Therefore, it is essential to provide a quantitative and understandable measurement to make the therapist’s diagnosis more objective. Several sensory systems can be used to trace hand motion, which can be categorized as camera-based, glove-based, magnetic actuator-based and inertial measurement unit (IMU)-based. Camera-based systems can be divided into two different types. One uses high-speed cameras to trace markers attached to body segments, which is quite accurate and often used as the reference [8]. However, occlusion problems will influence its accuracy and the distance between cameras and hands needs to be below a few meters in order to accurately measure hand and finger orientations. Because of these problems, you need many cameras (6 to 12). The other camera-based system traces objects, including their orientations, by exploiting depth maps to reconstruct the object [9,10]. Its advantage is that no finger or hand attachments are needed, making it friendly to users. However, this system also suffers from the occlusion problem and only allows hand movements to be evaluated if they occur in the vicinity of the cameras [11,12]. Besides, it requires a powerful processor to process the images. Glove-based sensor systems exploit varying sensors, such as resistive-bend sensors and optical-fiber sensors on the glove, transducing finger movement into corresponding signals to estimate relative orientations between hand and finger segments [13,14]. It has the benefit of a low price. However, the glove needs to be well attached for the measurement and requires thorough calibration before utilization. The magnetic actuator-based system also has two types, active actuation and passive actuation. The first one deploys active magnetic actuators on the finger tip and receivers on the dorsal side of the hand [15]. It has high accuracy and no occlusion issue. However, it requires different frequencies for each degree of freedom (DoF) of the actuator, which often needs equipment such as multiple power signal sources and a high-speed processor. This affects the complexity of the system and its physical dimensions. The second one uses magnets as passive sources, magnets are placed on the finger tips while magnetic sensors are worn on the wrist [16]. It has the benefit of having a simple structure and a low cost. However, it is difficult to distinguish the fields of different magnets, since only the sum of the fields are measured, especially when the magnets get close. The IMU-based system utilizes inertial sensors to trace the hand [17,18,19]. Compared with previous methods, it can provide raw data including angular velocity and acceleration. Orientation can be estimated by fusing the raw data. This operation suffers from drift, since it involves integration operations. However, this drift can be compensated using magnetometer data, which is easily accessible since it is often embedded in IMU systems. However, magnetometers are used in this solution and are therefore vulnerable to external magnetic disturbances, such as indoor iron surroundings [20,21]. Thomas and Wolfgang et al. proposed magnetometer-free methods for the joint angle estimation [22,23,24,25]. However, such methods assume the rotation is restricted to two DoFs because of the anatomy constraint [23]. Thus, they cannot be applied to flexible joints, such as the metacarpophalangeal joint (MCP) of the thumb. Relative orientations between the hand and its fingers are important for the reconstruction of hand-finger movement, which is essential information for AR, VR and rehabilitation.

Our goal is to estimate relative 3D orientations between finger tips and the dorsal side of the hand with only IMUs, essentially getting rid of magnetic disturbance by not using magnetometers. In order to reduce the integration drift, we exploit information during the daily life rather than using the biomechanical constraint. The information is based on the assumption that there are many instances in which hand and finger tips move together, experiencing almost the same angular velocities and accelerations represented in different 3D coordinate systems. The method was verified with a small sensor configuration: one sensor on the dorsal side of hand, and one on the most distal finger segment of interest.

## 2. Methods

In order to estimate the relative orientation, the information from the gyroscope and accelerometer and extra information during DEs need to be combined in an optimal way. Therefore, an extended Kalman filter (EKF) was introduced to estimate 3D relative orientations between the dorsal side of the hand and finger tips, assuming angular velocities and accelerations are the same, but just represented in a different coordinate system. The process model is based on integrating relative angular velocity, the measurement model is mainly based on the information during the DE. The quality of the DE is considered in the measurement variance. When the DE is available with small variance, we trust the measurement model more; otherwise, we trust the process model more. Thus, the information from process and measurement models is optimally fused to estimate relative 3D orientations during functional hand and finger movements.

### 2.1. Sensor Model

The gain error and non-orthogonality error are assumed to be time-invariant and can be obtained through sensor calibration; thus, the outputs of calibrated gyroscope can be expressed as

(1) $$\left\{\begin{array}{c} y_{gyr,h}^{h}=\omega_{h}^{h}+b_{h}+\zeta_{h}\\ y_{gyr,f}^{f}=\omega_{f}^{f}+b_{f}+\zeta_{f} \end{array}\right.$$

where $y_{gyr,h}^{h}$ and $y_{gyr,f}^{f}$ are gyroscope outputs on the hand and finger tip in their own frames. $b_{x}\left(x=h,f\right)$ is the slowly varying offset. $\zeta_{x}\left(x=h,f\right)$ is Gaussian noise.

For the calibrated accelerometer, the outputs on the hand and finger tips are

(2) $$\left\{\begin{array}{c} y_{acc,h}^{h}=a_{h}^{h}+g^{h}+\eta_{h}^{h}\\ y_{acc,f}^{f}=a_{f}^{f}+g^{f}+\eta_{f}^{f} \end{array}\right.$$

where g is the gravity, and $\eta_{h}^{h}$ and $\eta_{f}^{f}$ are Gaussian noise.

### 2.2. Process Model

The process model is based on integrating the relative angular velocity between the hand and its fingers in its own frame. We choose the quaternion $q_{hf}={\left[\begin{array}{cccc} q_{0} & q_{1} & q_{2} & q_{3} \end{array}\right]}^{T}$ that expresses relative orientation from a finger tip to the dorsal side of the hand as the state vector $x=q_{hf}$. The relative orientation $x_{k}$ is updated as

(3) $$x_{k}=x_{k-1}⊗\left[\begin{array}{cc} 1 & \frac{1}{2}\omega_{k}d_{t} \end{array}\right]+m$$

where m is the process error. $\omega_{k}$ is the relative angular velocity between the hand and fingers; ⊗ represents the multiplication operator between two quaternions.

(4) $$\omega_{k}={\left(\omega_{h}^{h}\right)}_{k}-x_{k-1}⊗{\left(\omega_{f}^{f}\right)}_{k}⊗x_{k-1}^{*}$$

where $\omega_{h}^{h}$ and $\omega_{f}^{f}$ are hand and finger angular velocities.

### 2.3. Measurement Model

The measurement update of EKF is based on the DE. During the DE, the hand and fingers share the same angular velocity in different coordinate frames

(5) $$\omega_{h}^{h}=q_{hf}⊗\omega_{f}^{f}⊗q_{hf}^{*}$$

where $\omega_{x}^{y}\left(x=h,f,y=h,f\right)$ is the angular velocity of an object in frame *x* expressed in the coordinate frame of object *y*. *h* represents the hand and *f* represents the finger tip. Combining Equations (1) and (5), we find:

(6) $$\begin{array}{ll} y_{gyr,h}^{h} & =q_{hf}⊗y_{gyr,f}^{f}⊗q_{hf}^{*}+b_{h}-q_{hf}⊗b_{f}⊗q_{hf}^{*}+\\ & \zeta_{h}-q_{hf}⊗\zeta_{f}⊗q_{hf}^{*}\\ & =q_{hf}⊗y_{gyr,f}^{f}⊗q_{hf}^{*}+d_{gyr} \end{array}$$

where the combined error of gyroscope $d_{gyr}$ is

(7) $$d_{gyr}=\left(b_{h}-q_{hf}⊗d_{f}⊗q_{hf}^{*})+(\zeta_{h}-q_{hf}⊗\zeta_{f}⊗q_{hf}^{*}\right)$$

Unlike the angular velocity, accelerations at different positions are different, which can be expressed as

(8) $$\begin{array}{c} a_{h}^{h}=q_{hf}⊗a_{f}^{f}⊗q_{hf}^{*}+\omega_{h}^{h}\times \left(\omega_{h}^{h}\times r_{fh}^{h}\right)+{\dot{\omega }}_{h}^{h}\times r_{fh}^{h}\\ =q_{hf}⊗a_{f}^{f}⊗q_{hf}^{*}+\left({\left\lfloor \omega_{h}^{h}\right\rfloor }_{\times }{\left\lfloor \omega_{h}^{h}\right\rfloor }_{\times }+{\left\lfloor {\dot{\omega }}_{h}^{h}\right\rfloor }_{\times }\right)r_{fh}^{h} \end{array}$$

where $a_{x}^{y}\left(x=h,f,y=h,f\right)$ is the acceleration of object in frame *x* relative to frame *y*. ${\dot{\omega }}_{h}^{h}$ is the hand angular acceleration in its own frame. $r_{fh}^{h}$ is the position vector between hand and fingers in the hand frame. ${\left\lfloor \right\rfloor }_{\times }$ denotes a skew-symmetric matrix.

(9) $${\left\lfloor a\right\rfloor }_{\times }=\left[\begin{array}{ccc} 0 & -a_{z} & a_{y}\\ a_{z} & 0 & -a_{x}\\ -a_{y} & a_{x} & 0 \end{array}\right]$$

If the second term $\left({\left\lfloor \omega_{h}^{h}\right\rfloor }_{\times }{\left\lfloor \omega_{h}^{h}\right\rfloor }_{\times }+{\left\lfloor {\dot{\omega }}_{h}^{h}\right\rfloor }_{\times }\right)r_{fh}^{h}$ is relatively small compared with the first term $q_{hf}⊗a_{f}^{f}⊗q_{hf}^{*}$, then Equation (8) can be approximated as the following equation:

(10) $$a_{h}^{h}\approx {q_{hf}⊗a}_{f}^{f}⊗q_{hf}^{*}$$

Combining Equations (2) and (8), we find:

(11) $$y_{acc,h}^{h}=q_{hf}⊗y_{acc,f}^{f}⊗q_{hf}^{*}+\left({\left\lfloor \omega_{h}^{h}\right\rfloor }_{\times }{\left\lfloor \omega_{h}^{h}\right\rfloor }_{\times }+{\left\lfloor {\dot{\omega }}_{h}^{h}\right\rfloor }_{\times }\right)r_{fh}^{h}+\eta_{c}$$

where the combined error $\eta_{c}$ can be expressed as

(12) $$\eta_{c}=\eta_{h}^{h}-q_{hf}⊗\eta_{f}^{f}⊗q_{hf}^{*}$$

Finally, an overall relation between hand and fingers based on Equations (6) and (11) is

(13) $$\left\{\begin{array}{c} y_{gyr,h}^{h}=q_{hf}⊗y_{gyr,f}^{f}⊗q_{hf}^{*}+d_{gyr}\\ y_{acc,h}^{h}=q_{hf}⊗y_{acc,f}^{f}⊗q_{hf}^{*}+\left({\left\lfloor \omega_{h}^{h}\right\rfloor }_{\times }{\left\lfloor \omega_{h}^{h}\right\rfloor }_{\times }+{\left\lfloor {\dot{\omega }}_{h}^{h}\right\rfloor }_{\times }\right)r_{fh}^{h}+\eta_{c} \end{array}\right.$$

Subsequently, we can get the measurement model based on the sensor model and quaternion constraint

(14) $$y_{k}=f\left(x_{k}\right)+v$$

where y and *f* can be expressed as

(15) $$y_{k}={\left[\begin{array}{ccc} {\left(y_{acc,h}^{h}\right)}^{T} & {\left(y_{gyr,h}^{h}\right)}^{T} & 0 \end{array}\right]}^{T}$$

(16) $$f\left(x\right)=\left[\begin{array}{c} x_{k}⊗y_{acc,f}^{f}⊗x_{k}^{*}\\ x_{k}⊗y_{gyr,f}^{f}⊗x_{k}^{*}\\ q_{0}^{2}+q_{1}^{2}+q_{2}^{2}+q_{3}^{2}-1 \end{array}\right]$$

As shown in Equations (3) and (16), the process and measurement model are both nonlinear with respect to $x_{k}$. In order to update the covariance matrix for $x_{k}$, linearization is performed and the Jacobian matrix F and H for process and measurement model are calculated; the details can be found in the Appendix A.

### 2.4. Uncertainty Error Variance

In order to assess the relative confidence in the measurement model (based on our DE assumptions) and the process model, the measurement variance is determined. According to the assumption that a hand and finger share approximately the same angular velocity and acceleration based on Equation (13), the differences in angular velocity and acceleration between the hand and fingers measured by the IMU determine the measurement variance. From Equation (7), the error is related to the offset error, the white noise and relative orientation. $d_{gyr}$ can be expressed with following equation from Equation (6).

(17) $$d_{gyr}=y_{gyr,h}^{h}-q_{hf}⊗y_{gyr,f}^{f}⊗q_{hf}^{*}$$

We approximate the distribution of $d_{gyr}$ as Gaussian distribution with zero mean and standard deviation $\sigma_{g}\left[\begin{array}{ccc} 1 & 1 & 1 \end{array}\right]$(rad/s)

(18) $$\sigma_{g}={\|y_{gyr,h}^{h}-q_{hf}⊗y_{gyr,f}^{f}⊗q_{hf}^{*}\|}_{2}$$

For Equation (13), the error $d_{acc}$ can be expressed with the following equation:

(19) $$d_{acc}=\left({\left\lfloor \omega_{h}^{h}\right\rfloor }_{\times }{\left\lfloor \omega_{h}^{h}\right\rfloor }_{\times }+{\left\lfloor {\dot{\omega }}_{h}^{h}\right\rfloor }_{\times }\right)r_{fh}^{h}+\eta$$

We can express the error in another format from Equation (11).

(20) $$d_{acc}=y_{acc,h}^{h}-q_{hf}⊗y_{acc,f}^{f}⊗q_{hf}^{*}$$

Similarly to the gyroscope, we assume the error $d_{acc}$ has an approximate Gaussian distribution with zero mean while its standard deviation $\sigma_{a}\left[\begin{array}{ccc} 1 & 1 & 1 \end{array}\right]$ is

(21) $$\sigma_{a}={\left\|y_{acc,h}^{h}-q_{hf}⊗y_{acc,f}^{f}⊗q_{hf}^{*}\right\|}_{2}$$

Based on the Gaussian approximation, as described in Equations (17) and (20), it is essential to know the rotation quaternion $q_{hf}$ before we get the variance. However, $q_{hf}$ is the variable we try to estimate which is also unknown. As we assume there is no or a slow orientation change between the hand and finger tips, the estimated relative orientation at time k−1 is used as the true relative orientation at time *k*.

(22) $$q_{hf,k}={\hat{q}}_{hf,k-1}$$

where $q_{hf,k}$ is the “true” rotation quaternion we use to estimate the variance at time *k*. ${\hat{q}}_{hf,k-1}$ is the estimated rotation quaternion at time k−1. The measurement covariance is determined as

(23) $$R_{m}=\left[\begin{array}{ccc} \sigma_{g}I_{3\times 3} & 0 & 0\\ 0 & \sigma_{a}I_{3\times 3} & 0\\ 0 & 0 & 0 \end{array}\right]$$

The initial value for the state vector of relative orientations $x_{k}$ was set as ${\left[\begin{array}{cccc} 1 & 0 & 0 & 0 \end{array}\right]}^{T}$.

## 3. Experiments

### 3.1. Experiment Setup

**Figure 1:**
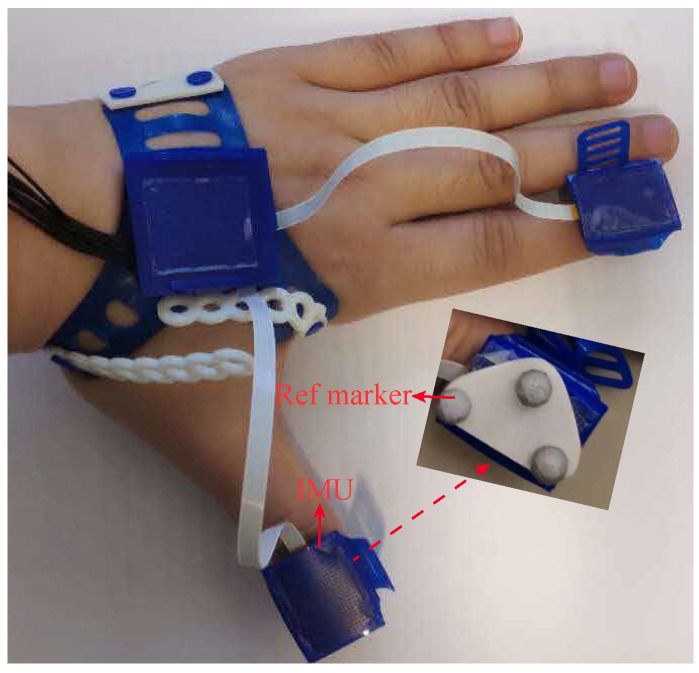
IMUs on the dorsal side of the hand and fingertips. The inset shows the cluster of optical markers used on top of each IMU for reference measurement of segment orientations using the optical VICON system. Every cluster contains three markers, which determine a 3D coordinate frame.

The sensor system includes three IMUs fixed on the most distal segments of the thumb and index finger and the dorsal side of the hand, as shown in Figure 1. MPU9250 (InvenSense) was chosen for the IMU, which contains a tri-axis accelerometer and tri-axis gyroscope (it also contains a tri-axis magnetometer, which was not used in the current study). All IMUs were sampled synchronously; the sample frequencies of gyroscope and accelerometer were 200 Hz and 100 Hz respectively. All the data were collected by a master micro-controller (Atmel XMEGA) and then transmitted to the PC via a USB connection. Prior to the experiment, the accelerometer was calibrated based on local gravity; the gyroscope was calibrated based on the calibrated accelerometer [26]. An optical Vicon system with eight cameras was used to perform 3D orientation reference measurements. For this purpose, three optical markers were attached to each IMU. The sampling frequency of Vicon was 100 Hz.

### 3.2. Alignment of the IMU and Reference Marker Frame for the Validation Experiment

For evaluation of the IMU-based 3D relative orientation estimation using the optical system, it is essential to calibrate the relative orientation between the sensor and marker-based reference frame. Here, we used the accelerometer for this marker system’s IMU calibration. Holding the system static, we obtained the gravity in the IMU frame from the accelerometer readings. Meanwhile, we obtained the orientation from the global Vicon frame to marker frame $q_{mg}$. Gravity in marker frame is $q_{mg}⊗g⊗q_{mg}^{*}$, where g is gravity in global Vicon frame (*z*-axis of global Vicon system was vertical upward; gravity in this frame was $g=\left[\begin{array}{ccc} 0 & 0 & -g \end{array}\right]$; *g* is the local gravity value). When we have at least two poses, we obtain more than two vectors expressed in the marker frame and IMU frame respectively, which is enough to determine the relative orientation between the IMU and marker frame.

### 3.3. Sensor to Segment Calibration

Before the experiment, IMU errors were calibrated according to D Tedaldi et al.’s and WT Fong et al.’s research [26,27], including sensitivity error, offset error, non-orthogonal error and misalignment between the accelerometer and gyroscope. After the IMU was fixed on the hand and fingers, the relative orientations between IMU sensors and body segments were calibrated. An accelerometer was used to achieve the alignment by exploiting static accelerometer measures of gravity. When we held our hand sequentially horizontally and vertically, we obtained the 3D relative orientation between two frames. More details can be found in Kortier et al.’s research [28].

### 3.4. Synchronization of Vicon and IMU System

In this experiment, the two measurement systems were synchronized by recording the sensed responses of an induced impact at the start and end of each experiment. At the start and end of every experiment, we hit the IMU on a desk, resulting in an acceleration peak measured by the IMU system and a minimum vertical position of the Vicon markers simultaneously, which was used to synchronize the two systems.

### 3.5. Protocols for the Experiment

**Figure 2:**
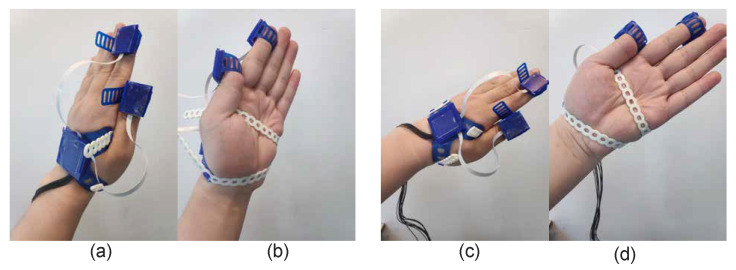
Movement for task 1: rotations of the hand, while not varying relative orientations between the hand and fingers. Subfigure (**a**,**b**) are a set of pronation and supination movements. Subfigure (**c**,**d**) are another set of pronation and supination but with a different rotation axis. During this task, we did the pronation and supination movements with different rotation axes.

In order to demonstrate the feasibility of our approach, an experimental part was designed to estimate the accuracy of the algorithm compared with the optical system. Our feasibility experiment involved three participants. The protocol was reviewed, approved and conducted under the auspices of the Ethics Committee EEMCS, Univerisity of Twente. The following tasks were performed:

*Task1: Movements and rotations of the hand, while not varying relative orientations between hand and fingers*: IMUs were fixed on fingers and the dorsal side of the hand. Then, the participant did the pronation and supination movements with the arm while the axis of pronation and supination was continuously changing. The orientation was changed over approximately 160° around the rotation axis; see Figure 2. Furthermore, we varied the angular velocity by performing these cyclical movements with varying repetition rate of pronation and supination (60, 120, 240 cycles/min), with the help of a metronome. This was done in order to test the performance of the algorithm under different conditions. During the process, the subject was asked to close the hand and not change the relative orientations between the hand and fingers, while displacing or rotating the hand.

**Figure 3:**
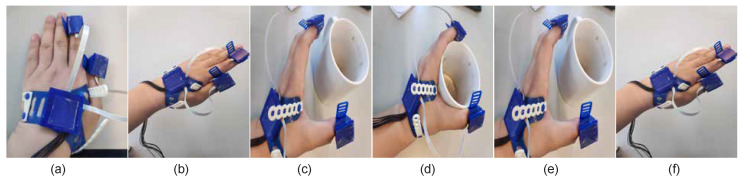
Movement for task 2: Simple functional task. The task can be divided into several phases. (**a**) Put the hand static on the desk; (**b**) raise the hand; (**c**) grasp the cup; (**d**) drink the water; (**e**) release the hand; (**f**) withdraw the hand.

*Task2: Simple functional task*. The subject was asked to place the hand on the desk; then rise the hand and grasp a cup; subsequently drink some water and place the cup back; and finally place the hand on the original position. The illustration of the movement can be seen in Figure 3.

For task 1, the orientation reference was directly derived from the IMUs, because the relative orientation was imposed by the hand, and therefore, known and not varying. For task 2, the reference measurement was performed using the optical VICON system (software version 2.8.2).

## 4. Results

### 4.1. Movements and Rotations of the Hand, While Not Varying Relative Orientations between the Hand and Finger (Task 1)

**Figure 4:**
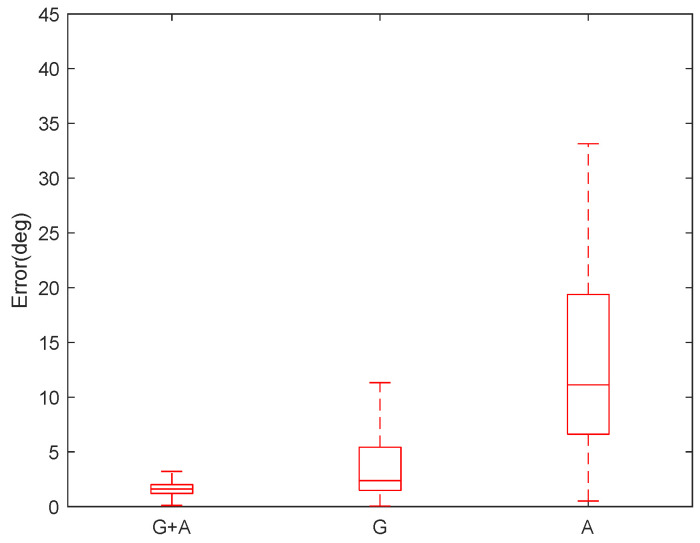
Estimated orientation error $|\theta_{err}|$ with gyroscope and accelerometer (values under 99.3 percent coverage are shown in the boxplot figures). “G”, “A” and “G+A” represent estimated results based on gyroscope, accelerometer and gyroscope plus accelerometer respectively.

The error angle used was the arccos of the first component of quaternion error $q_{err}$ [29]:

(24) $$q_{err}=q_{est}^{-1}⊗q_{ref}=\left[\begin{array}{cc} 1 & \frac{1}{2}\theta_{err} \end{array}\right]$$

where $q_{est}$ was the estimated relative orientation and $q_{ref}$ was the orientation reference. We obtained more than two independent vectors from the gyroscope, accelerometer or both from 3D movements. The error angle estimated when DE is available is shown in Figure 4. The orientation error is smallest with the gyroscope and accelerometer, while the orientation error is largest with accelerometer data only.

#### Influence of Repetition Rate of Movement

**Figure 5:**
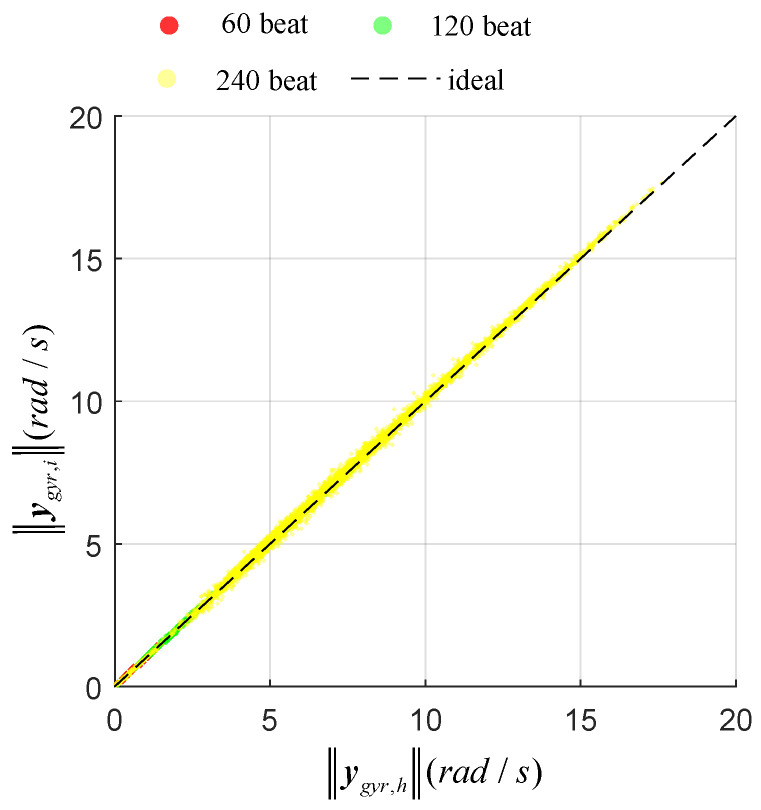
Relation of output norms between gyroscopes on the dorsal side of the hand and finger tip with different repetition rates.

**Figure 6:**
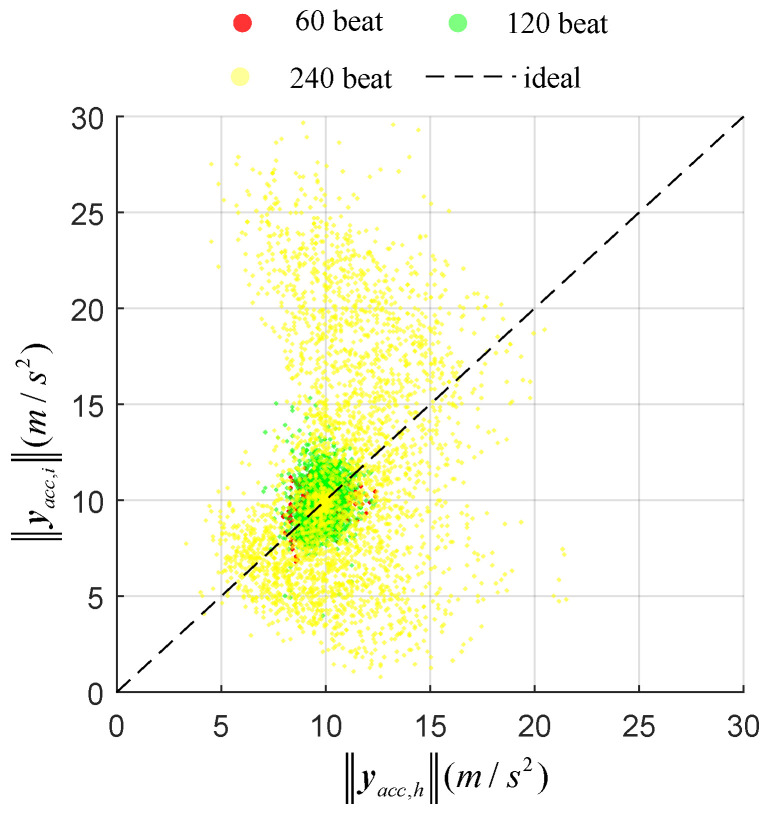
Relation of output norms between accelerometers on the dorsal side of the hand and finger tip with different repetition rates.

**Figure 7:**
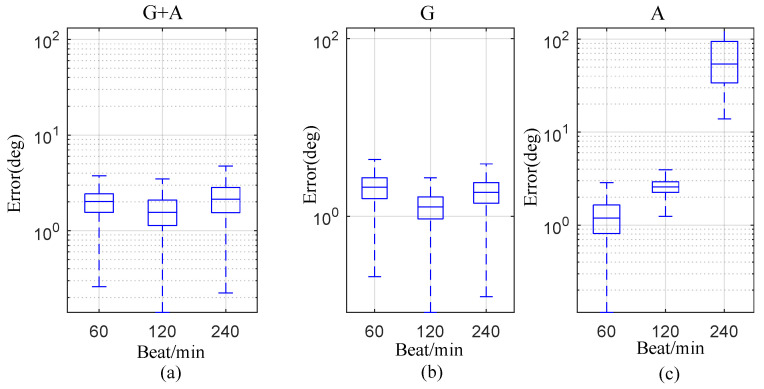
Estimation error $|\theta_{err}|$ with different repetition rates (values under 99.3 percent coverage are shown in the boxplot figures). Subfigures (**a**–**c**) are estimations with gyroscope plus accelerometer, and gyroscope and accelerometer individually.

The estimation may be influenced by the repetition rate of movements. Figure 5 and Figure 6 show the relation between the norm of gyroscope or accelerometer on thte hand and finger for several repetition rates. Ideally, the gyroscope output norms $\|y_{gyr,h}\|$, $\|y_{gyr,f}\|$ should be equal for the measurement update and for the accelerometer. The differential output norms cause estimation errors, as shown in Equation (13). For the accelerometer, the different output norms $\left|\right\|y_{acc,h}\left\|-\right\|y_{acc,f}\left\|\right|$ were 29.3 m/s${}^{2}$, 66.4 m/s${}^{2}$ and 370.2 m/s${}^{2}$ under the repetition rates 60, 120 and 240 beats/min respectively. Meanwhile, the correspondingly differential output norms of gyroscope were 2.2 rad/s, 2.7 rad/s and 4.4 rad/s. As shown in Figure 7b,c, the estimated orientation error based on the accelerometer became larger when the repetition rate increased, while orientation error based on gyroscope changed little when the repetition rate increased. As shown in Figure 7a, the estimated result based on the gyroscope and accelerometer trusted the gyroscope more than the accelerometer because it contained less error; thus, it was also insensitive to repetition rate.

### 4.2. Simple Functional Task (Task 2)

**Figure 8:**
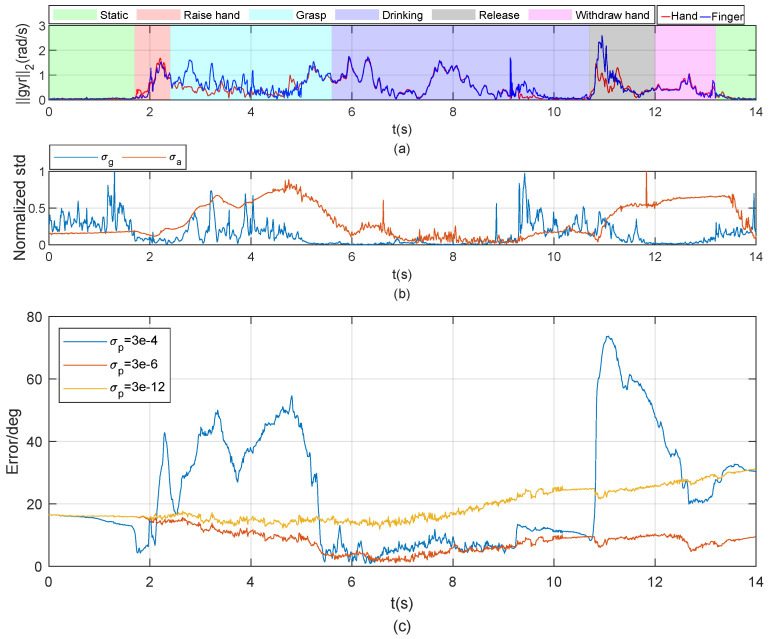
Relative orientation between hand and thumb during the water-drinking process. Subfigure (**a**) shows the output norms of the two gyroscopes (on the hand and finger tip respectively). Subfigure (**b**) shows the normalized SDs $\sigma_{a}$ and $\sigma_{g}$ from Equations (18) and (21). Larger $\sigma_{a}$ and $\sigma_{g}$ mean larger measurement error. The EKF trusts the process model more and the measurement model less when $\sigma_{a}$ and $\sigma_{g}$ are larger. Subfigure (**c**) shows the estimated results with different SDs of the process model. The variance of process error Q was determined as $\sigma_{p}I_{4}$.

**Figure A1:**
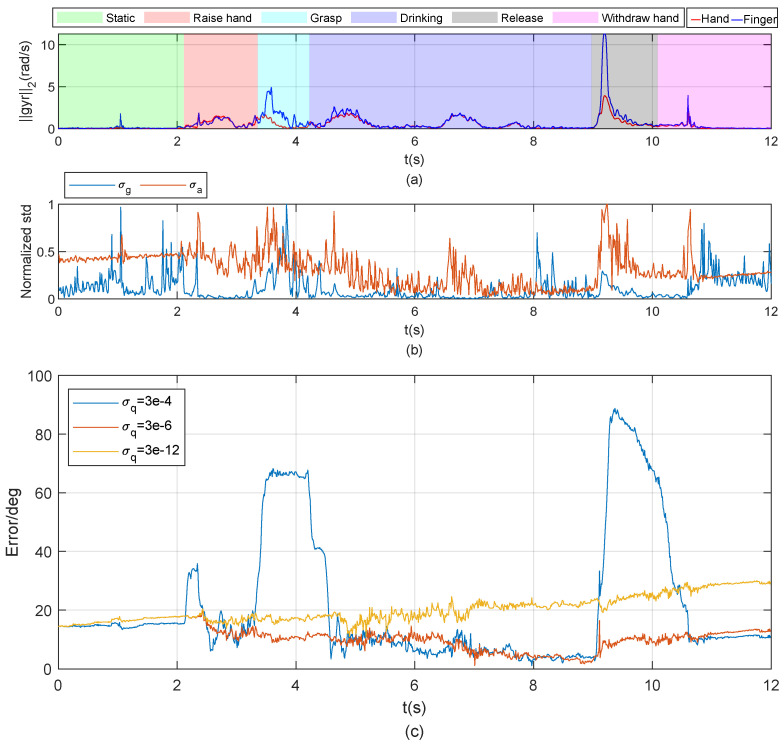
Relative orientation between the hand and thumb during the water-drinking process. Subfigure (**a**) shows the output norms of the two gyroscopes (on the hand and finger tip respectively). Subfigure (**b**) shows the normalized SDs $\sigma_{a}$ and $\sigma_{g}$ from Equations (18) and (21). Larger $\sigma_{a}$ and $\sigma_{g}$ mean larger measurement error. The EKF trusts the process model more and the measurement model less when $\sigma_{a}$ and $\sigma_{g}$ are larger. Subfigure (**c**) shows the estimated results with different SDs of the process model. The variance of process error Q was determined as $\sigma_{p}I_{4}$.

**Figure A2:**
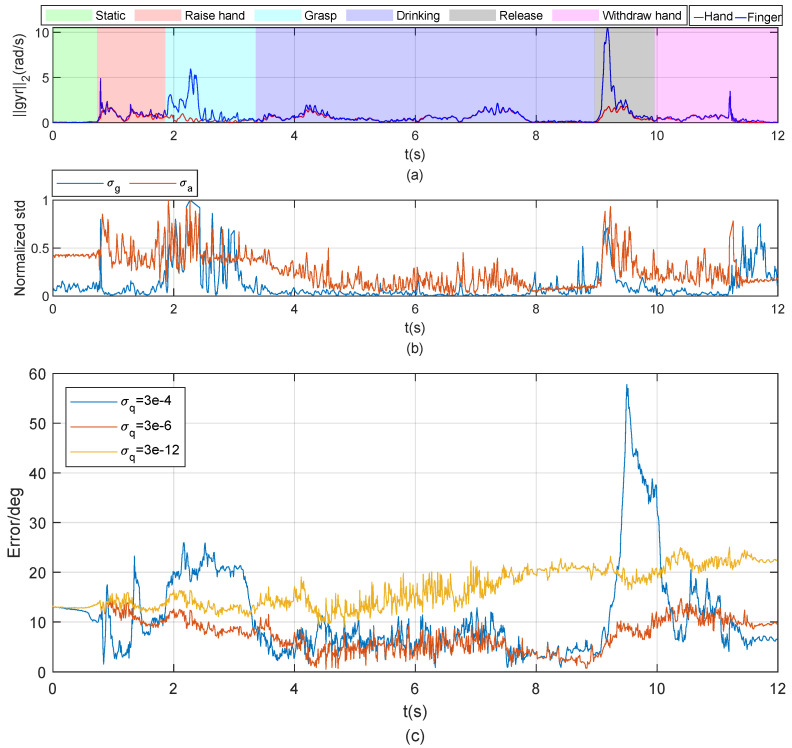
Relative orientation between the hand and thumb during the water-drinking process. Subfigure (**a**) shows the output norms of the two gyroscopes (on the hand and finger tip respectively). Subfigure (**b**) shows the normalized SDs $\sigma_{a}$ and $\sigma_{g}$ from Equation (18) and (21). Larger $\sigma_{a}$ and $\sigma_{g}$ mean larger measurement error. The EKF trusts the process model more and the measurement model less when $\sigma_{a}$ and $\sigma_{g}$ are larger. Subfigure (**c**) shows the estimated results with different SDs of the process model. The variance of process error Q was determined as $\sigma_{p}I_{4}$.

**Figure A3:**
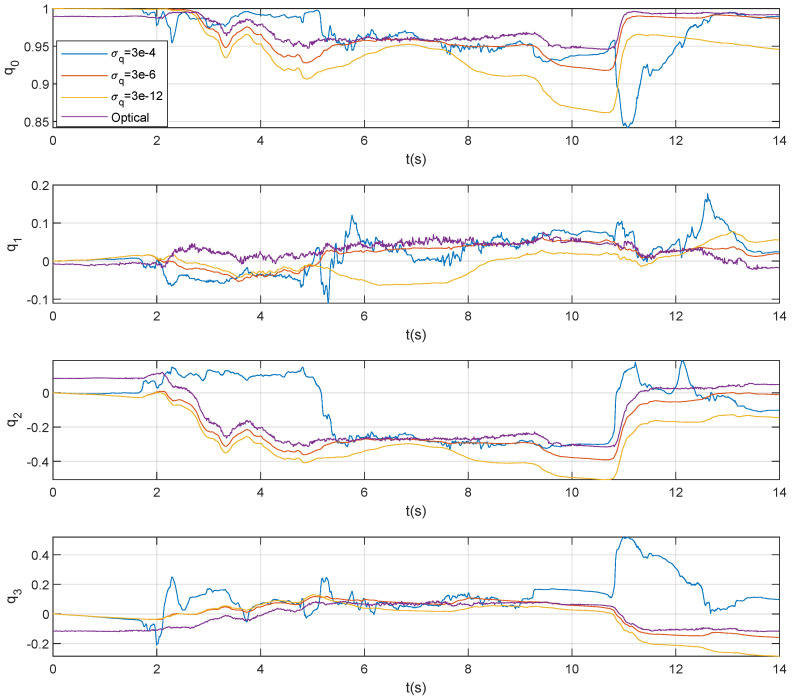
Estimation based on relative orientation between the hand and thumb based on IMU system and optical system. Orientations are expressed based on quaternion; based on these results, we obtained the orientation error in subfigure (c) of Figure 8. $\sigma_{p}$ has the same meaning as in Figure 8.

According to Figure 3, the whole process was divided into several phases; the estimated orientation errors based on the optical system in different phases are shown in Figure 8. The quaternion-based orientation estimated by IMU system and optical can be seen in Figure A3 in the Appendix B. The error during the drinking part was relatively low because the cub imposed a constant relative orientation on the hand and fingers and the whole hand moved with varying position and orientation, as shown in Figure 8. Since the angular velocity and acceleration norms were close to each other, the standard deviations of measurement noise $\sigma_{a}$ and $\sigma_{g}$ were small, as shown in subfigure (b); the measurement model was trusted relatively more relative to the process model under said condition. For the other phases of this functional task, there were bigger differences between gyroscope and accelerometer norms on the hand and fingers; thus $\sigma_{a}$ and $\sigma_{g}$ were bigger; the trust in the process model was relatively high. A good estimation of relative orientation was achieved by choosing a suitable standard deviation for the process error (see Figure 8c). The results of other two participants can be seen in Figure A1 and Figure A2 in the Appendix B.

## 5. Discussion

We proposed and evaluated an IMU-based setup for estimating 3D relative orientation between hand and finger tips. Compared with the IMU-based data glove system described by Salchov-Homer et al. [19] and Kortier et al. [28], we reduced the number of IMUs as much as possible and avoided magnetic disturbance, but still obtained comparable precision of estimated orientation. In reference [19], the orientation error magnitude is approximately five to ten degrees. In our research, the orientation error is related to the movement quality. When the hand and fingers move together, the median orientation error can be smaller than five degrees. For the water-drinking experiment, the estimated error is less than ten degrees when hand and fingers approximately move together, but around ten degrees during the rest periods. In our view, this is a promising method for the hand finger orientation estimation with a small IMU configuration which can be used if rich whole-hand movements occur and the change of relative orientations between hand and finger tips is regular and relatively small. Standard deviations $\sigma_{g}$ and $\sigma_{a}$ can be used to assess whether such DEs regularly apply during a specify movement.

Most previous IMU-based systems [17,28] for finger orientation estimation usually require a magnetometer to reduce the drift caused by the gyroscope, which will suffer from the magnetic disturbance problem in indoor environments. To our knowledge, in order to remove magnetic disturbance but still suppress the drift, a biomechanical model is additionally used in methods described in the literature (e.g., [17,28]). We have not applied additional information from a bioimechanical model in our current study, although this additional information could be applied. However, it should be noted that finger movements are usually assumed to be restricted to two DoFs while using biomechanical constraints. In construct, our method can be implemented without biomechanical constraints and can be applied to estimating three-DoF-relative orientation during 3D hand movements without such biomechanical assumptions.

For the result in task 1, the relative orientation estimation is less sensitive to an increase of repetition rate of the same movement when using gyroscopes or gyroscopes plus an accelerometer than the accelerometer only. That is because as the difference among the accelerometer signals from the hand and finger becomes larger, the non-gravitational acceleration caused by increasing angular velocity or angular acceleration becomes relatively more important compared to the gravity component.

Position estimation only based on inertial sensors is quite challenging and limited by integration drift. Our further research will concentrate on relative position estimation based on IMUs combined with sensing the magnetic field of a magnetic source. For this to be feasible, an adequate estimate of relative orientation is required, so the 3D magnetic field measurement can be expressed in the coordinate system of the magnetic source. This is an essential first step in estimating relative positions. In this research, only one healthy participant was involved since we are mainly concentrating on verifying the performance of the algorithm. Subsequently, the proposed relative orientation and position estimation methods for the hand and finger using a small sensing configuration need to be evaluated in healthy subjects and patients during more complex daily tasks, in order to assess the applicability in clinical and daily-life settings. To make the system more friendly to users, the system could be wireless in the future.

## 6. Conclusions

In conclusion, IMUs can be used to estimate the relative orientation between the hand and fingers without using magnetometers. Compared with previous systems, we only exploit IMUs on finger tips and the dorsal side of the hand rather than having IMUs on every segment. The performance is dependent on how well the hand and fingers move together, which influences the accuracy of the estimate. The median value of estimation error can be smaller than five degrees when IMUs are on our hand and fingers if their relative orientation is not variant over time, while the object or hand is moving. During the water-drinking task, the estimation error can be smaller than 10 degrees during periods when the hand and fingers approximately move together, which may be adequately accurate to provide useful information to clinicians when judging.

## Appendix A

By linearizing the nonlinear function of the process model and measurement model, we obtained the Jacobian matrixes F and H respectively, which were used in EKF for the covariance update. Based on Equation (3):

(A1) $$\begin{array}{cc} F & =\frac{\partial x_{k}}{\partial x_{k-1}}=\frac{1}{2}\left[\begin{array}{cccc} a_{11}\omega_{2} & a_{12}\omega_{2} & a_{13}\omega_{2} & a_{14}\omega_{2}\\ a_{21}\omega_{2} & a_{22}\omega_{2} & a_{23}\omega_{2} & a_{24}\omega_{2}\\ a_{31}\omega_{2} & a_{32}\omega_{2} & a_{33}\omega_{2} & a_{34}\omega_{2}\\ a_{41}\omega_{2} & a_{42}\omega_{2} & a_{43}\omega_{2} & a_{44}\omega_{2} \end{array}\right]d_{t}\\ \; & +\frac{1}{2}\left[\begin{array}{cccc} 0 & -\omega_{1x} & -\omega_{1y} & -\omega_{1z}\\ \omega_{1x} & 0 & \omega_{1z} & -\omega_{1y}\\ \omega_{1y} & -\omega_{1z} & 0 & \omega_{1x}\\ \omega_{1z} & \omega_{1y} & -\omega_{1x} & 0 \end{array}\right]d_{t}+I_{4\times 4} \end{array}$$

(A2) $$\left\{\begin{array}{cc} a_{11}=2\left[\begin{array}{ccc} q_{0}q_{1} & q_{0}q_{2} & q_{0}q_{3} \end{array}\right] & a_{12}=\left[\begin{array}{ccc} 1+2q_{1}^{2} & 2q_{1}q_{2} & 2q_{1}q_{3} \end{array}\right]\\ a_{13}=\left[\begin{array}{ccc} 2q_{1}q_{2} & 1+2q_{2}^{2} & 2q_{2}q_{3} \end{array}\right] & a_{14}=\left[\begin{array}{ccc} 2q_{1}q_{3} & 2q_{2}q_{3} & 1+2q_{3}^{2} \end{array}\right]\\ a_{21}=-\left[\begin{array}{ccc} 1+2q_{0}^{2} & 2q_{0}q_{3} & -2q_{0}q_{2} \end{array}\right] & a_{22}=-2\left[\begin{array}{ccc} q_{0}q_{1} & q_{1}q_{3} & -q_{1}q_{2} \end{array}\right]\\ a_{23}=\left[\begin{array}{ccc} -2q_{0}q_{2} & -2q_{2}q_{3} & 1+2q_{2}^{2} \end{array}\right] & a_{24}=-\left[\begin{array}{ccc} 2q_{0}q_{3} & 1+2q_{3}^{2} & -2q_{2}q_{3} \end{array}\right]\\ a_{31}=-\left[\begin{array}{ccc} -2q_{0}q_{3} & 1+2q_{0}^{2} & 2q_{0}q_{1} \end{array}\right] & a_{32}=-\left[-2\begin{array}{ccc} q_{1}q_{3} & 2q_{0}q_{1} & 1+2q_{1}^{2} \end{array}\right]\\ a_{33}=-2\left[\begin{array}{ccc} -q_{2}q_{3} & q_{0}q_{2} & q_{1}q_{2} \end{array}\right] & a_{34}=\left[\begin{array}{ccc} 1+2q_{3}^{2} & -2q_{0}q_{3} & -2q_{1}q_{3} \end{array}\right]\\ a_{41}=-\left[\begin{array}{ccc} 2q_{0}q_{2} & -2q_{0}q_{1} & 1+2q_{0}^{2} \end{array}\right] & a_{42}=\left[\begin{array}{ccc} -2q_{1}q_{2} & 1+2q_{1}^{2} & -2q_{0}q_{1} \end{array}\right]\\ a_{43}=-\left[\begin{array}{ccc} 1+2q_{2}^{2} & -2q_{1}q_{2} & q_{0}q_{2} \end{array}\right] & a_{44}=-2\left[\begin{array}{ccc} q_{2}q_{3} & -q_{1}q_{3} & q_{0}q_{3} \end{array}\right] \end{array}\right.$$

Based on Equation (16):

(A3) $$\begin{array}{cc} \; & H=\frac{\partial f}{\partial x_{k}}=\left[\begin{array}{c} \frac{\partial (x_{k}⊗y_{acc,f}^{f}⊗x_{k}^{*})}{\partial x_{k}}\\ \frac{\partial (x_{k}⊗y_{gyr,f}^{f}⊗x_{k}^{*})}{\partial x_{k}}\\ \frac{\partial (q_{0}^{2}+q_{1}^{2}+q_{2}^{2}+q_{3}^{2})}{\partial x_{k}} \end{array}\right]\\ \; & =2\left[\begin{array}{cccc} b_{11}y_{acc,f}^{f} & b_{12}y_{acc,f}^{f} & b_{13}y_{acc,f}^{f} & b_{14}y_{acc,f}^{f}\\ b_{21}y_{acc,f}^{f} & b_{22}y_{acc,f}^{f} & b_{23}y_{acc,f}^{f} & b_{24}y_{acc,f}^{f}\\ b_{31}y_{acc,f}^{f} & b_{32}y_{acc,f}^{f} & b_{33}y_{acc,f}^{f} & b_{34}y_{acc,f}^{f}\\ b_{11}y_{gyr,f}^{f} & b_{12}y_{gyr,f}^{f} & b_{13}y_{gyr,f}^{f} & b_{14}y_{gyr,f}^{f}\\ b_{21}y_{gyr,f}^{f} & b_{22}y_{gyr,f}^{f} & b_{23}y_{gyr,f}^{f} & b_{24}y_{gyr,f}^{f}\\ b_{31}y_{gyr,f}^{f} & b_{32}y_{gyr,f}^{f} & b_{33}y_{gyr,f}^{f} & b_{34}y_{gyr,f}^{f}\\ q_{0} & q_{1} & q_{2} & q_{3} \end{array}\right] \end{array}$$

(A4) $$\left\{\begin{array}{cc} b_{11}=\left[\begin{array}{ccc} q_{0} & q_{3} & -q_{2} \end{array}\right] & b_{12}=\left[\begin{array}{ccc} q_{1} & q_{2} & q_{3} \end{array}\right]\\ b_{13}=\left[\begin{array}{ccc} -q_{2} & q_{1} & -q_{0} \end{array}\right] & b_{14}=\left[\begin{array}{ccc} -q_{3} & q_{0} & q_{1} \end{array}\right]\\ b_{21}=\left[\begin{array}{ccc} -q_{3} & q_{0} & q_{1} \end{array}\right] & b_{22}=\left[\begin{array}{ccc} q_{2} & -q_{1} & q_{0} \end{array}\right]\\ b_{23}=\left[\begin{array}{ccc} q_{1} & q_{2} & q_{3} \end{array}\right] & b_{24}=\left[\begin{array}{ccc} -q_{0} & -q_{3} & q_{2} \end{array}\right]\\ b_{31}=\left[\begin{array}{ccc} q_{2} & -q_{1} & q_{0} \end{array}\right] & b_{32}=\left[\begin{array}{ccc} q_{3} & -q_{0} & -q_{1} \end{array}\right]\\ b_{33}=\left[\begin{array}{ccc} q_{0} & q_{3} & -q_{2} \end{array}\right] & b_{34}=\left[\begin{array}{ccc} q_{1} & q_{2} & q_{3} \end{array}\right] \end{array}\right.$$

## Appendix B

In this section, three figures are shown. Figure A1 and Figure A2 are the results of task 2 from other two participants. Compared with the first participant, the drinking phase (see subfigure (d) of Figure 3) is replaced as displacing, which means, we did not put the cup to the mouth but to another position on the desk. Figure A3 is the estimation of relative orientation between the hand and fingers based on the IMU and optical system; based on this, we obtained the orientation error in subfigure (c) in Figure 8.
